# Severe Pneumonia Associated with Pandemic (H1N1) 2009 Outbreak, San Luis Potosí, Mexico

**DOI:** 10.3201/eid1601.090941

**Published:** 2010-01

**Authors:** Alejandro Gómez-Gómez, Martin Magaña-Aquino, Christian A. García-Sepúlveda, Uciel R. Ochoa-Pérez, Reynaldo Falcón-Escobedo, Andreu Comas-García, Saray Aranda-Romo, Hugo I. Contreras-Treviño, Paulina V. Jiménez-Rico, Mario A. Banda-Barbosa, Félix Dominguez-Paulin, J. Mario Bernal-Blanco, Luis F. Pérez-González, Daniel E. Noyola

**Affiliations:** Hospital Central “Dr. Ignacio Morones Prieto,” San Luis Potosí, Mexico (A. Gómez-Gómez, M. Magaña-Aquino, R. Falcón-Escobedo, J.M. Bernal-Blanco, L.F. Pérez-González); Universidad Autónoma de San Luis Potosí, San Luis Potosí (A. Gómez-Gómez, M. Magaña-Aquino, C.A. García-Sepúlveda, U.R. Ochoa-Pérez, R. Falcón-Escobedo, A. Comas-García, S. Aranda-Romo, H.I. Contreras-Treviño, P.V. Jiménez-Rico, M.A. Banda-Barbosa, F. Dominguez-Paulin, J.M. Bernal-Blanco, L.F. Pérez-González, D.E. Noyola); Servicios de Salud del Estado de San Luis Potosí, San Luis Potosí (U.R. Ochoa-Pérez)

**Keywords:** Influenza, respiratory infections, adult respiratory distress syndrome, acute lung injury, pneumonia, pandemic, H1N1, expedited, Mexico, research, *Suggested citation for this article*: Gómez-Gómez A, Magaña-Aquino M, García-Sepúlveda CA, Ochoa-Pérez UR, Falcón-Escobedo R, Comas-García A, et al. Severe pneumonia associated with pandemic (H1N1) 2009 outbreak, San Luis Potosí, Mexico. Emerg Infect Dis [serial on the Internet]. 2010 Jan [*date cited*]. Available from http://www.cdc.gov/EID/content/16/1/27.htm

## Abstract

We describe the clinical characteristics and outcomes of adults hospitalized with pneumonia during the pandemic (H1N1) 2009 outbreak. Patients admitted to a general hospital in San Luis Potosí, Mexico, from April 10 through May 11, 2009, suspected to have influenza virus–associated pneumonia were evaluated. We identified 50 patients with suspected influenza pneumonia; the presence of influenza virus was confirmed in 18: 11 with pandemic (H1N1) 2009 virus, 5 with unsubtypeable influenza A virus, 1 with seasonal influenza A virus (H3N2), and 1 in whom assay results for seasonal and pandemic (H1N1) 2009 viruses were positive. Eighteen patients were treated in the intensive care unit, and 10 died. During the pandemic (H1N1) 2009 outbreak, severe pneumonia developed in young adults who had no identifiable risk factors; early diagnosis and treatment of influenza virus infections may have a determinant role in outcome.

## Abstract 2

Severe pneumonia developed in young adults who had no identifiable risk factors.

A novel influenza A virus, pandemic (H1N1) 2009 virus, has been identified as the cause of an epidemic outbreak of respiratory illness throughout the world (*1*). Current information indicates that the pandemic (H1N1) 2009 virus has been circulating in Mexico since at least March 2009 and that it has been the cause of an increase in the number of hospitalizations of young adults since April 2009 (*1*). The city of San Luis Potosí played a major role in raising awareness of the presence of an unusual and nonsubtypeable influenza A virus during the early days of the outbreak (*2*).

The Hospital Central “Dr. Ignacio Morones Prieto” is a public hospital located in San Luis Potosí that served as a reference center for evaluation and treatment of patients with suspected influenza virus pneumonia. In this article, we describe the results of investigations performed to determine the etiology of this outbreak and report the clinical findings, treatments, and patient outcomes.

## Patients and Methods

### Study Site

The city of San Luis Potosí is located in central Mexico and is the capital of the state of San Luis Potosí (*3*). The state population was 2,410,414 in 2005; the population of the San Luis Potosí metropolitan area in the same year was 957,753 (*4*). The Hospital Central provides medical services to mid- and low-income populations from all areas of the state; it has 269 beds in total, 250 in adult and pediatric wards and 19 in the intensive care unit (ICU).

### Patients

Starting April 10, 2009, when the first case of pandemic (H1N1) 2009 was identified, all patients admitted to Hospital Central with lower respiratory tract infections (LRTIs) were screened for the presence of influenza virus. Patients who experienced acute onset of respiratory symptoms (cough, rhinorrhea, and/or dyspnea) and fever were assessed for possible influenza-related pneumonia, including radiologic evaluation. This report includes all adult patients with radiographic evidence of pneumonia admitted during the first month of the pandemic (H1N1) 2009 outbreak.

### Clinical, Laboratory, and Bacteriologic Information

Patients admitted to medical wards and the ICU were subjected to the hospital’s standard diagnostic protocol: blood cultures, HIV antibody testing, chest radiograph, laboratory tests, and spontaneous or induced sputum samples for Gram staining and culture. These tests were performed at admission and every 48–72 hours thereafter. Patients receiving mechanical ventilation had samples for minibronchoalveolar lavage (mini-BAL) (*5*), blood, and urine sent for bacterial cultures at admission and every 48–72 hours, or in the event of new or persistent fever. Bronchoscopy with BAL was performed as clinically indicated. A quantitative threshold of 10^5^ CFU/mL for mini-BAL was used and of 10^4^ CFU/mL for BAL (*6*). Patients were treated with at least the following standard antimicrobial drug regimens: those with criteria for acute lung injury (ALI, partial pressure of oxygen in arterial blood [paO_2_]/fraction of inspired oxygen [FiO_2_] = 201–300) or acute respiratory distress syndrome (ARDS, paO_2_/FiO_2_<200) received ceftriaxone and/or vancomycin plus levofloxacin or clarithromycin, and patients treated in medical wards received levofloxacin. Therapeutic decisions were individualized by the attending physician. Deceased patients for whom the family granted consent were subjected to postmortem examinations to recover lung specimens for bacteriologic culture, influenza virus detection, and histopathologic analysis.

### Sample Collection and Processing

Respiratory samples obtained from patients with suspected influenza infection were placed in either normal saline or viral transport media and kept refrigerated at 4ºC until their arrival at the laboratory. A 200-µL aliquot of these samples was used for RNA extraction, and the remaining volume was stored at –70°C. Viral RNA was isolated using the High Pure Viral RNA Kit (Roche Diagnostics, Mannheim, Germany). Sample collection and processing were carried out according to national and international biosafety guidelines and recommendations (*7**,**8*).

### Influenza Virus Detection

A multiplex reverse transcription–PCR (RT-PCR) capable of distinguishing type A from type B influenza virus was developed based on previously published primers (*9*). One-step RT-PCR was performed using the Access RT-PCR System (Promega Corporation, Madison, WI, USA) at 48ºC for 45 min, followed by 94°C for 2 min, 40 cycles of 30 s at 94°C, 30 s at 56°C, and 30 s at 72°C, and a final step at 72ºC for 5 min. Influenza A samples were subsequently subtyped using a multiplex PCR with sequence specific primers approach adapted from previous publications (Table 1) (*11*). PCR products were subjected to electrophoresis on 1.5% agarose gels prestained with ethidium bromide at 6 volts/cm for 45 min and digitally documented. An RT-PCR for detection of pandemic (H1N1) 2009 virus was developed based on early sequencing data (EpiFlu Database, http://platform.gisaid.org). RT was carried out at 38ºC for 45 min by using UniFlu-RT oligonucleotide; PCR involved the newly developed Sw-NS-F 5′-ATG-GAC-TCC-AAC-ACC-3′ and Sw-NS-R 5′-TTA-AAT-AAG-CTG-AAA-CGA-G-3′ oligonucleotides at 1.6 µmol/L final concentration, 0.02 IU/µL of Taq (Vivantis Technologies Sdn Bhd Selangor D.E., Malaysia), 200 µmol/L deoxynucleoside triphosates, and 1.5 mmol/L MgCl_2_. PCR program was 95ºC for 5 min, followed by 15 cycles of 95ºC for 20 s, 54ºC for 30 s, and 72ºC for 1.5 min, and a final step of 2 min at 72ºC. One microliter of a 1:1 dilution of the first PCR product was then used in a second PCR by using similar components except 1 mmol/L MgCl_2_ and 800 nmol/L final concentration of internal oligonucleotides Sw1-F 5′-CTT-GAA-AGA-GGA-ATC-GAG-CG-3′ and Sw1-R 5′-GTC-TCC-CAT-TCT-CAT-CAC-AGT-3′ (429-bp amplicon). Influenza virus detection at the Instituto de Diagnóstico y Referencia Epidemiológicos (InDRE) and the State Public Health Laboratory used the real-time RT-PCR protocol developed by the US Centers for Disease Control and Prevention (CDC).

**Table 1:** Oligonucleotide primers used for the subtyping of type A influenza viruses, Mexico

Oligonucleotide	Sequence (5′ → 3′)	Amplicon, bp	Reference
UniFlu-RT	AGC-AAA-AGC-AGG	Full genomic	(*10*)
HA1-F	GAA-ATT-TGC-TAT-GGC-TGA-CGG-GR	171	This study
HA1-R	GAC-ACT-ACA-GAG-ACA-TAA-GCA-TTT-TC		(*11*)
HA3-F	CAG-CAA-AGC-CTA-CAG-CAA-MTG-TT	236	This study
HA3-R	GGC-ATA-GTC-ACG-TTC-AAT-GCT-G		(*11*)
HA5-F	AAA-CTC-CAA-TRG-GGG-CGA-TAA-AC	344	This study
HA5-R1	CAA-CGG-CCT-CAA-ACT-GAG-TGT		(*11*)
HA5-R2	CCA-ACA-GCC-TCA-AAC-TGA-GTG-T		This study
NA1Nw-F	ACT-CAR-GAG-TCT-GAA-TGT-G	409	This study
NA1Nw-R1	GTC-CTT-CCT-ATC-CAA-ACA-CC		
NA1Nw-R2	GTT-CTC-CCG-AGC-CAG-ATA-CC		
NA2-F1	GGA-AAA-TCG-TTC-ATA-CTA-GCA-MAT-TG	176	This study
NA2-F2	GGG-AAA-ATC-GTT-CAT-ATT-AGC-ACA-TTG		(*11*)
N2-R1	AGC-ACA-CAT-AWC-TGG-AAA-CAA-TGC		This study

### Virologic and Epidemiologic Data

To assess the presence of respiratory syncytial virus (RSV) and influenza in the community, we reviewed the database at the Virology Laboratory Universidad Autónoma de San Luis Potosí (UASLP) and recorded the weekly number of viral detections from January 2008 through May 2009. In addition, the weekly number of acute respiratory infections (ARI) reported in the state of San Luis Potosí and the emergency department (ED) at the Hospital Central was recorded.

### Statistical Analysis

We analyzed demographic and clinical characteristics using descriptive statistics. Means or medians were used for description of continuous variables according to data distribution; categorical variables were described with the use of percentages. Comparisons among patient groups were made to assess factors associated with severe infections. We compared categorical variables using the χ^2^ or Fisher exact test; continuous variables were compared by using the Student *t* test or Mann-Whitney U test, according to data distribution. A p value <0.05 was considered significant. Statistical analyses were performed with SPSS for Windows (version 14.0, SPSS Inc., Chicago, IL, USA).

## Results

An ARI outbreak was recorded in San Luis Potosí during April and May 2009. The most characteristic feature observed at the beginning of this outbreak was an increase in severe pneumonia cases requiring hospitalization of young adult patients. The Figure shows the epidemiologic curve of the weekly number of ARI cases reported to Servicios de Salud en el Estado de San Luis Potosí (state public health services) from January 2008 through May 2009 and the weekly number of ARI-related consultations provided in the ED at Hospital Central. In addition, the percentage of samples that were positive for influenza and RSV during each week at the Virology Laboratory UASLP is presented. Pneumonia patients included in this report were admitted during epidemiologic weeks 14 through 18 at a time that maximal pandemic (H1N1) 2009 virus circulation was documented.

**Figure:**
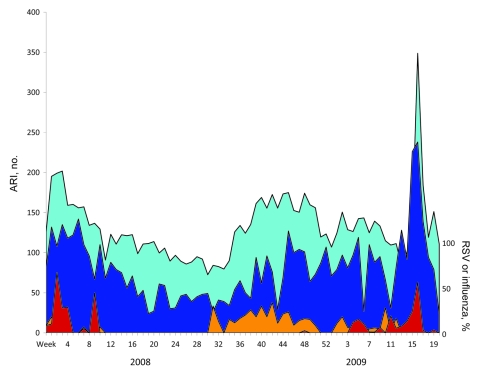
Weekly number of acute respiratory infections (ARI) reported in the state of San Luis Potosí, Mexico (no. of cases × 100, light blue area); weekly number of ARI visits at the emergency department of Hospital Central “Dr. Ignacio Morones Prieto” (dark blue area); and weekly percentage of samples positive for respiratory syncytial virus (RSV; orange area) or influenza (red area), Virology Laboratory, Universidad Autónoma de San Luis Potosí, during January 2008 through May 2009.

From April 10 through May 11, 2009, a total of 70 patients >18 years of age were admitted to the Hospital Central with suspected LRTI. After review of clinical, radiologic, and laboratory data, suspected influenza virus–related pneumonia was diagnosed for 50 of those patients; other patients were determined to have cholangitis and ARDS (1 patient), congestive heart failure (2 patients), aspiration pneumonia (2 patients), asthma without evidence of pneumonia on chest radiograph (5 patients), and influenza-like syndrome without pneumonia (10 patients). Nine of the 20 excluded patients were tested for influenza infection, and results were negative for 8; samples from 1 of the patients found to have influenza-like illness without radiographic evidence of pneumonia were positive for pandemic (H1N1) 2009 virus.

The main demographic features of our group of 50 patients are summarized in Table 2. Only 1 patient had an underlying pulmonary disorder. Obesity was the most frequent underlying disorder and was the only underlying disorder in 17 patients; 6 patients had morbid obesity (body mass index [BMI] >40). The most frequent symptoms patients had at hospital admission were fever (100%), headache (96%), cough (94%), and myalgias (94%). The predominant radiologic pattern on chest radiograph was consolidation, with multilobar and unilobar consolidation in 39 and 11 patients, respectively. Twenty-one patients had ill-defined interstitial opacities. Three patients had evidence of unilateral pleural effusion that affected ≈10% of the hemithorax. HIV testing was performed for 45 patients; all results were negative. Evidence of a systemic inflammatory response and muscle involvement was observed commonly, manifested by elevated levels of C-reactive protein, creatine phosphokinase (CPK), lactate dehydrogenase (LDH), and aspartate aminotransferase (AST).

**Table 2:** Demographic features of 50 adult patients admitted with acute pneumonia during the pandemic (H1N1) 2009 outbreak, San Luis Potosí, Mexico*

Characteristic	Value
Age, y, mean (SD, range)	38.4 (13.9, 21–69)
Gender	
M	29 (58)
F	21 (42)
Residence	
San Luis Potosí municipality	30 (60)
Other municipality	20 (40)
No. home contacts, median (range)	4 (1–16)
Presence of >1 underlying condition	30 (60)
Obesity (BMI > 30)	25 (55.6)†
Diabetes mellitus	8 (16)
Other conditions‡	9 (18)
Smoker	13 (26)
Arrival at the hospital	
Directly	40 (80)
Referred from other hospital or clinic	10 (20)
Influenza vaccination during previous winter season	5 (10)

*Values are no. (%) unless otherwise indicated. BMI, body mass index. †BMI data available only for 45 patients. ‡Some patients had >1 additional underlying condition, including chronic renal failure (3), underlying heart disorder (3), systemic lupus erythematosus (1), chronic obstructive lung disease (1), hyperthyroidism (1), seizure disorder (1), and pregnancy (1).

Thirty patients were treated in medical wards, 12 of whom met criteria for ALI, none had extrapulmonary involvement, and all were discharged to home. Two patients with ARDS were treated in the ER.

Eighteen patients were treated in the ICU (17 with ARDS and 1 with ALI). All patients with ARDS were mechanically ventilated. The median positive end expiratory pressure at admission was 16 cm H_2_O (range 14–20 cm) and media FiO_2_ was 80% (range 50%–100%); the mean Sepsis-related Organ Failure Assessment (SOFA) score and Acute Physiology and Chronic Health Evaluation (APACHE) II at admission were 9.44 (range 5–15) and 17 (range 8–32), respectively. Twelve patients had hemodynamic instability that required vasoconstrictors and 6 had arrhythmias, mainly supraventricular and ventricular extrasystoles. Acute renal failure developed in 7 patients (6 within 72 hours of admission); 6 of these 7 patients experienced hemodynamic instability, and 3 required hemodialysis. Nine patients with evidence of cardiac failure underwent transthoracic echocardiography. The mean left ventricular ejection fraction was 54.6%; in 2 patients, it was reduced at 40%. Nosocomial pneumonia developed in 8 patients in the ICU after a median of 9.5 days of mechanical ventilation.

Forty-five patients received antiviral treatment with oseltamivir. The time elapsed from onset of symptoms to the start of antiviral drug treatment ranged between 1 and 14 days (median 6 days). Four patients that did not receive antiviral drugs were discharged. The only patient who did not receive antiviral drugs and died was the first patient with pandemic (H1N1) 2009–associated pneumonia to be admitted to our hospital during this outbreak; he had severe pneumonia and ARDS and died in the ED.

All patients were treated with antimicrobial drugs. Levofloxacin or clarithromycin was included as part of the antimicrobial drug treatment of 48 (96%) patients; the 2 patients who did not receive 1 of these antimicrobial drugs recovered and were discharged to home. Sixteen patients received steroids as part of their treatment; steroid use was assessed by the attending physician. The median number of days from admission to start of steroid treatment was 2 days (range 0–20 days). The effect of steroid use on patient outcome is difficult to assess because this treatment was used more frequently in patients with more severe disease.

Postmortem open lung biopsies were performed on 5 patients and a complete necropsy was performed for 1. The pathologic findings were those of diffuse alveolar damage; additionally, some patients had hemophagocytosis and capillary and arteriolar thrombosis as well as enlargement of alveolar cells. One patient had acute bacterial pneumonia.

Respiratory samples from 45 patients were available for viral detection. Samples from 37 patients were tested at UASLP, while samples from 41 patients were tested at InDRE in Mexico City or the State Public Health Laboratory in San Luis Potosí using the real time RT-PCR protocol developed by CDC. Overall, influenza virus was detected in respiratory secretions of 15 (33.3%) of the 45 patients who had respiratory samples available for testing; pandemic (H1N1) 2009 virus was detected for 10 patients, seasonal influenza A virus was detected for 4, and results of assays for pandemic (H1N1) 2009 and seasonal influenza A viruses were positive for 1 patient. Subtyping of samples positive for seasonal influenza virus showed that 1 infection was caused by influenza A (H1N1) virus (this finding corresponded to the patient with positive results for both seasonal and pandemic [H1N1] 2009 virus; due to the potential for cross-reactivity, infection with pandemic [H1N1] 2009 virus only cannot be ruled out in this patient) and 1 by influenza A (H3N2); the other 3 samples were not subtypeable by our RT-PCR. In addition, RT-PCR was performed in lung tissue from 6 patients who died; the presence of influenza virus was detected in 3 of these patients (pandemic [H1N1] 2009 virus in 1 and unsubtypeable influenza virus in 2). Detection of influenza virus in respiratory samples from these deceased patients was either negative (in 2 patients) or not possible because no respiratory sample was collected before the patient’s death (1 patient).

The duration of symptoms at the time of sample collection for samples that tested positive for influenza virus at UASLP was significantly shorter (mean of 4.7 days) compared with those samples that tested negative (8.8 days; p = 0.02). Duration of symptoms at the time of sample collection was also demonstrated to be statistically significant for samples that tested positive with the use of CDC’s real time RT-PCR protocol (mean 5.7 days) compared with those that were negative by this method (mean 9.5 days; p = 0.006).

We compared the demographic and clinical characteristics of patients with pandemic (H1N1) 2009 infections and those that were negative on viral testing (Table 3). The clinical presentation of both groups of patients was similar.

**Table 3:** Demographic, clinical, and laboratory features on admission of patients with pandemic (H1N1) 2009 and nonconfirmed influenza pneumonia cases during the pandemic (H1N1) 2009 outbreak, San Luis Potosí, Mexico*

Characteristic	Pandemic (H1N1) 2009 (n = 11)	Nonconfirmed influenza (n = 32)	p value
Demographic features			
Male sex	5 (45.5)	20 (62.5)	0.48
Age, y, mean (SD)	34 (10.97)	39.47 (14)	0.25
Resident of San Luis Potosí municipality	5 (45.5)	20 (62.5)	0.73
Clinical features			
Presence of underlying conditions			
Obesity (BMI >30)†	4 (40)	16 (57.1)	0.47
Diabetes	2 (18.2)	4 (12.5)	0.64
Other conditions‡	3 (27.3)	4 (12.5)	0.35
Days from symptom onset to admission, mean (SD)	5.36 (2.5)	6.72 (3.13)	0.2
Duration of symptoms at the onset of dyspnea, d, mean (SD)	2.73 (1.19)	3.91 (1.92)	0.06
Pneumonia severity index (PORT score)	2.82 (1.47)	2.81 (1.15)	0.99
Symptoms			
Fever	11 (100)	32 (100.0)	NA
Headache	10 (90.9)	31 (96.9)	0.45
Cough	11 (100)	31 (96.9)	1
Myalgias	9 (81.8)	32 (100.0)	0.06
Arthralgias	10 (90.9)	30 (93.8)	1
Dyspnea	11 (100)	25 (78.1)	0.16
Rhinorrea	8 (72.7)	25 (78.1)	0.69
Malaise	6 (54.5)	11 (34.4)	0.29
Blood-streaked sputum	3 (27.3)	13 (40.6)	0.49
Diarrhea	1 (9.1)	7 (21.9)	0.66
Pleuritic chest pain	1 (9.1)	5 (15.6)	1
Vomiting	2 (18.2)	2 (6.3)	0.27
Physical examination findings on admission			
BMI, mean (SD)	29.17 (6.48)	31.22 (5.4)	0.34
Respiratory rate, bpm, mean (SD)	28.44 (4.91)	28.31 (7.24)	0.92
Crackles			0.03
Unilateral	4 (36.4)	2 (6.3)	
Bilateral	7 (63.6)	30 (93.8)	
Wheezing	5 (45.5)	18 (56.3)	0.73
Laboratory features			
Total leukocyte count on admission (x 10^9^/L), median (SD)	8.81 (6.1)	6.81 (4.13)	0.23
Platelet count (x 10^9^/L), mean (SD)§	173.6 (61.48)	227 (216.24)	0.45
C-reactive protein, mg/dL, mean (SD)	14.51 (8.45)	18.74 (16.22)	0.41
Creatinine, mg/dL, median (SD)	1.24 (0.87)	1.11 (0.75)	0.13
Creatine phosphokinase, U/L, mean (SD)	376.78 (439.22)	462.2 (507.27)	0.65
Lactate dehydrogenase, U/L, median (SD)§	952 (674.72)	1089.31 (602.32)	0.54
AST, mg/dL, median (range)¶	43 (19–76)	69 (18–249)	0.07
ALT, mg/dL, median (range)#	29 (13–55)	43 (13–177)	0.05

*Values are no. (%) unless otherwise indicated. BMI, body mass index; bpm, breaths per minute; PORT, Pneumonia Patient Outcomes Research Team (www.ahrq.gov/clinic/pneuclin.htm); NA, not available; AST, aspartate aminotransferase; ALT; alanine transaminase. †Data available for 38 patients. ‡Includes chronic renal failure, systemic lupus erythematosus, pregnancy, cardiac disorder, seizure disorder. §Data available for 42 patients. ¶Data available for 41 patients. #Data available for 40 patients.

Sputum cultures obtained at time of admission were positive in 4 patients treated in medical wards; bacteria isolated included *Staphylococcus aureus* (2 patients) and *Streptococcus pneumoniae* (2 patients). For patients treated in the ICU all cultures performed on mini-BAL and BAL (1 patient) at admission were negative; however, nosocomial pneumonia developed in 8 patients; microorganisms isolated included *Acinetobacter baumanii* (5 patients), *S. aureus* (1 patient), *Escherichia coli* (1 patient) and *Candida* sp*.* (1 patient). BAL was performed for only 4 patients as part of evaluation of suspected nosocomial pneumonia. Six postmortem lung biopsy specimens were cultured; 1 showed growth of *E. coli*; the other 5 were negative.

Ten (20%) patients died; 8 were obese (BMI >30), and 4 of those were morbidly obese (BMI >40). Eight patients died in the ICU with a median length of stay of 8.5 days (range 2–14 days), and 2 patients with ARDS died in the ED within hours after arrival. Six patients died within the first 7 days following admission, and 4 died during the second week after admission. In 5 patients, the cause of death was multiorgan dysfunction (pulmonary, cardiovascular, and renal); 3 deaths were attributed to severe ARDS refractory to ventilator management protocols (*12*); 1 was caused by mixed ARDS and shock refractory to vasoconstrictors; and 1 was caused by septicemia. The median length of stay for patients treated in the ICU was 13 days (range 2–60 days); for those treated on the medical wards, this figure was 5 days (range 2–12 days).

The median duration of symptoms at time of admission for patients who died was longer (7.5 days) than for those who survived (5 days, p = 0.025); duration of symptoms before admission for patients who died in the ER was similar to duration of symptoms for patients who died in the ICU. The mean SOFA and APACHE II scores on admission for patients treated in the ICU who survived were 7.5 (range 5–11) and 13 (range 8–18), respectively; in comparison, for patients who died the mean values of these scores were 12 (range 10–15) and 21 (range 15–32), respectively. On the other hand, patients who received antiviral drug therapy during the first 5 days after initiation of symptoms had a better outcome than those who did not (survival of 95% of patients vs. 70%, respectively; p = 0.037). However, sample size was not sufficient to perform an appropriate multivariate analysis to define the independent contribution of duration of illness, antiviral drug therapy, and other variables to patient outcome.

## Discussion

Since pandemic (H1N1) 2009 was first identified in spring 2009, there has been a worldwide dissemination of this virus (*1*). Although the precise origin of this strain has not yet been determined, Mexico was the first country to show the effects of the epidemic caused by this virus. In addition, more severe cases have been registered in our country, particularly in young adults, in comparison to the rest of the world.

Pneumonia is recognized as the most important complication of influenza infections (*13*). LRTI in patients with influenza may be caused directly by viral invasion or result from bacterial complications (*14**,**15*). The occurrence of progressive disease, with bilateral pulmonary consolidation or ARDS, associated with high death rates has been described for patients with influenza-associated pneumonia, particularly in those with infections caused by new subtypes of influenza virus. In contrast, bacterial pneumonia complicating influenza usually occurs after near resolution of influenza symptoms (*16**,**17*). Our study cohort was composed mostly of young adults without severe cardiovascular or pulmonary underlying disorders in whom the onset of dyspnea was, on average, 3.5 days after the onset of influenza symptoms. In only 4 (8%) of the 50 patients included in this report was there clear evidence of the participation of bacteria in the disease process; this number contrasts with previous reports of community-acquired pneumonia, for which bacteria are reported more frequently. Our results suggest that bacterial co-infection played a minor role as a cause of pneumonia in these patients. In addition, no histopathologic evidence of bacterial pneumonia was observed in 5 of the 6 deceased patients on whom lung biopsies were performed.

Although we were not able to confirm pandemic (H1N1) 2009 virus in all patients, several factors support the notion that a high proportion of their infections were caused by this virus. These factors include the clustering of cases of severe pneumonia in young adults (>85% of the patients became ill during a 2-week period) coincident with the identification of a novel influenza virus shown to be circulating in the community.

A striking feature observed in this cohort of patients was the high frequency of systemic involvement with laboratory evidence of myositis/rhabdomyolysis, elevated acute-phase reactants, ARDS, hemodynamic instability, and acute renal failure. Elevation of LDH levels has been reported for patients with seasonal influenza but has not been considered a specific marker of severity. Elevation of CPK levels was frequently observed in patients treated at our hospital, and elevation of CPK and AST levels suggest that muscle inflammation was present in these patients. Myositis (with or without rhabdomyolysis) has been associated previously to influenza infections (*18**–**21*). Of note, elevated CPK levels were observed in patients with and without confirmed influenza virus infections.

Why these severe manifestations of influenza infections occurred in young adult patients during this outbreak remains an unanswered question. Although almost two thirds of patients hospitalized with pneumonia at our institution had underlying health disorders, the presence of chronic respiratory or cardiovascular diseases were rare. Obesity was the most frequent underlying disorder and was present in more than half of the patients; in contrast, the prevalence of obesity for adults >20 years of age in San Luis Potosí was 31.3% in 2006 (*22*). Eight of 10 patients who died were obese, including 4 who were morbidly obese. Obesity has been suggested to be a proinflammatory chronic condition and thus may have contributed to some of the manifestations observed in these patients (*23**,**24*). The presence of obesity as a possible risk factor for severe pandemic (H1N1) 2009 has been noted previously. In a report from Michigan, 9 of 10 patients with pandemic (H1N1) 2009 infection requiring intensive care were obese (*25*). However, larger studies are needed to determine the role of obesity as a risk factor for severe influenza pneumonia.

There are several limitations to our study. First, only pneumonia patients were included. Therefore, the effect of influenza as a cause of hospitalizations is probably underestimated because patients with exacerbations of chronic obstructive pulmonary disease and asthma, as well as with congestive heart failure decompensation triggered by influenza, may have been excluded. Second, we did not have appropriate respiratory samples from all patients admitted with pneumonia because some patients were admitted before the existence of an outbreak had been identified. In addition, patients with more severe infections tended to have a longer duration of illness at the time of admission, reducing the likelihood that influenza may have been detected. Third, influenza infection could not be confirmed in most patients, and other causes of acute pneumonia (such as other viruses) were not excluded. However, because the clinical manifestations were similar in all cases, patients became ill in a short period simultaneously with a sudden increase in ARI in San Luis Potosí, and pandemic (H1N1) 2009 infections were detected at a time when RSV and seasonal influenza circulation had declined, we consider it is likely that many cases may have been caused by influenza virus.

During the pandemic (H1N1) 2009 outbreak in San Luis Potosí, Mexico, young adults without identifiable risk factors became ill with severe pneumonia. Until predictors of severe pandemic (H1N1) 2009 virus infections are identified, early diagnosis and prompt antiviral treatment seem to be the best measures to avoid serious illness caused by this virus (*26**,**27*).
